# Chest CT in COVID-19 pneumonia: correlations of imaging findings in clinically suspected but repeatedly RT-PCR test-negative patients

**DOI:** 10.1186/s43055-021-00481-6

**Published:** 2021-04-06

**Authors:** Inan Korkmaz, Nursel Dikmen, Fatma Oztürk Keleş, Tayibe Bal

**Affiliations:** 1 Department of Radiology, Faculty of Medicine, Hatay Mustafa Kemal University, Hatay, Turkey; 2 Department of Chest Diseases, Faculty of Medicine, Hatay Mustafa Kemal University, Hatay, Turkey; 3 Department of Infectious Disease and Clinical Microbiology, Faculty of Medicine, Hatay Mustafa Kemal University, Hatay, Turkey

**Keywords:** Computed tomography, Polymerase chain reaction, Severe acute respiratory syndrome coronavirus 2, Viral load

## Abstract

**Background:**

To emphasize the importance of CT in the diagnosis of COVID-19 disease by comparing the thoracic CT findings of COVID-19 patients with positive RT-PCR results and patients with clinical suspicion of COVID-19 but with negative RT-PCR results.

**Results:**

In our study, COVID-19 patients with positive RT-PCR results (RT-PCR (+) group) and patients with clinical suspicion of COVID-19 but negative RT-PCR results (RT-PCR (−) group) were compared in terms of CT findings. In CT images, ground-glass opacity and ground-glass opacity + patchy consolidation were the most common lesion patterns in both groups. No statistically significant differences in the rates and types of lesion patterns were observed between the two groups. In both groups, lesion distributions and distribution patterns were similarly frequent in the bilateral, peripheral, and lower lobe distributions. Among the 39 patients who underwent follow-up CT imaging in the first or second month, a regression in lesion number and density was detected in 18 patients from both groups. Consolidations were completely resorbed in 16 of these patients, and five patients had newly developed fibrotic changes. The follow-up CT examination of 16 patients was normal.

**Conclusions:**

Due to the false-negative rate of RT-PCR tests caused by various reasons, clinically suspected COVID-19 patients with a contact history should be examined with CT scans, even if RT-PCR tests are negative. If the CT findings are positive, these patients should not be removed from isolation.

## Background

On December 31, 2019, the World Health Organization China Country Office reported cases of pneumonia of unknown etiology detected in Wuhan, China’s Hubei province. In January 2020, the Chinese authorities identified a new kind of coronavirus [1].

In the week of 7–13 September, 1.8 million new cases and more than 40,600 additional deaths were reported, which was still slightly increased compared to the previous week [2]. With Coronavirus disease 2019 (COVID-19) becoming a global threat, its clinical course and imaging findings are key aspects to identify affected patients [3, 4]. Therefore, early and accurate diagnostic tools for this disease have become important.

Real-time polymerase chain reaction (RT-PCR) remains the standard test of COVID-19 pneumonia but standby time for viral detection with RT-PCR tests, incomplete sampling techniques, variations in viral load, and false-negative rates of a test depending on the kit sensitivity can delay the diagnosis. Although the first test is negative in a number of cases, it has been reported that positivity develops in the second, third, or even subsequent tests [5–9]. There are also cases in the literature with multiple negative RT-PCR test results from nasopharyngeal samples that are positive in tests using bronchoalveolar lavage (BAL) samples. The target receptor of the virus is angiotensin-converting enzyme 2 (ACE2). This receptor is not expressed at the nasal and oral levels but substantially in type 1 and type 2 alveolar epithelial cells, making the BAL method more effective [10].

Although the symptoms of the disease may be similar to those of other viral infections, differences in imaging findings can facilitate the differential diagnosis [11]. Imaging techniques such as radiography and computed tomography (CT) have gained importance for disease detection [12]. Studies have been performed by radiologists to determine typical and atypical CT findings of COVID-19 infection, and radiologists have attempted to reach consensus regarding these findings. Common CT findings of COVID-19 pneumonia are mostly ground-glass opacity (GGO), consolidation and crazy-paving pattern, and less commonly subpleural curvilinear line, air bubble sign, halo and reversed halo sign, air bronchogram, airway changes, and fibrous stripe formations [13–16].

In this study, we aimed to emphasize the importance of CT in the diagnosis of COVID-19 disease by comparing the thoracic CT findings of COVID-19 patients with positive RT-PCR results and patients with clinical suspicion of COVID-19 but with negative RT-PCR results.

## Methods

This retrospective study was approved by the local ethics committee and the requirement for informed consent was waived (27/07/2020, meeting number 9, decision number 27).

### Patients

Patients suspected of having COVID-19 infection who were referred to our hospital were evaluated by the Chest Diseases and Infectious Diseases departments of our hospital. RT-PCR was performed with nasal and nasopharyngeal sampling in patients whose clinical symptoms and laboratory findings (lymphopenia and C-reactive protein, ferritin, blood sedimentation rate, and D-dimer elevation) were indicative of COVID-19 infection, those who had contact with COVID-19 patients, and those who had recently returned from abroad (Europe, Saudi Arabia).

From March to August 2020, a total of 211 patients suspected of having COVID-19 were evaluated. Eighty-one patients with a pathology other than COVID-19 infection, a known lung disease, inaccessible CT images, and no history of contact were excluded from the study. As a result, the data of 130 patients were examined in terms of RT-PCR and CT findings.

Patients whose initial RT-PCR test was positive or whose initial test was negative but became positive in subsequent tests were included in the RT-PCR (+) group, and these patients were considered as the COVID-19 patient group.

**Fig. 1:**
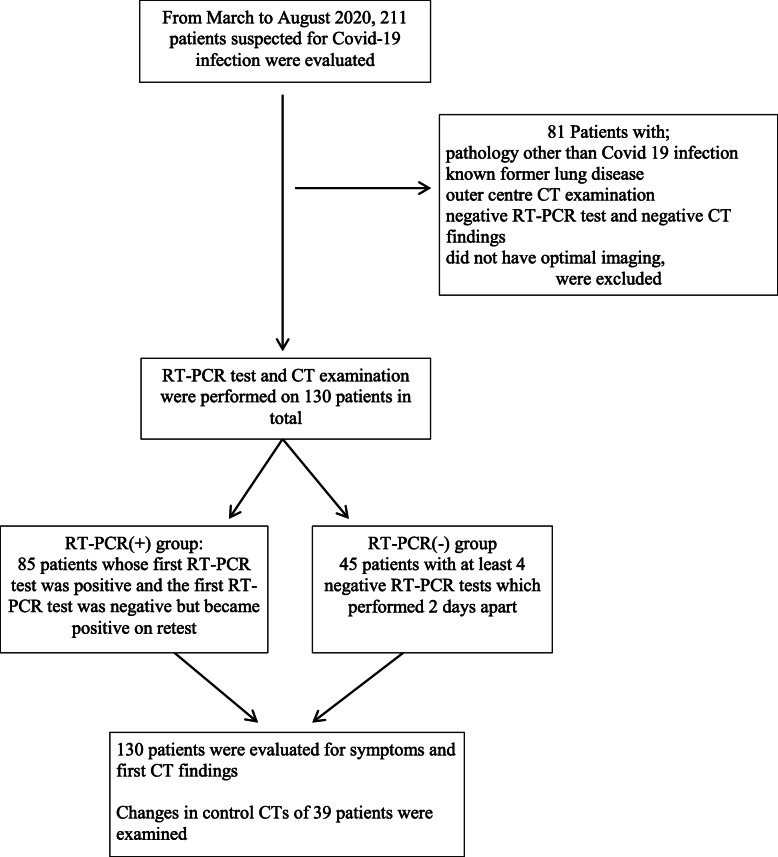
Flow diagram of the study

Patients with negative initial RT-PCR tests underwent at least four more tests every 2 days, and if all RT-PCR tests were negative, these patients were included in the RT-PCR (−) group. Patients in the RT-PCR (−) group either had a family member with COVID-19 disease or had direct contact with COVID-19 patients, or some had a recent travel history abroad. Thus, 85 RT-PCR (+) and 45 RT-PCR (−) patients were evaluated in terms of symptoms and CT findings (Fig. 1).

The patients were clinically examined in terms of symptoms such as fever, cough, shortness of breath, diarrhea, anorexia, abdominal pain, headache, chest pain, and inability to taste and smell.

### CT protocol

CT scans were performed in the supine position using either the Hitachi Eclos 16 (5 mm slice thickness, 120 kV, 75 mAs) or the 64-slice Toshiba Aquilion (5 mm slice thickness, 120 kV, 25 mAs) units. CT was performed with the patient in the supine position during end-inspiration. Multiplanar images were obtained using the multiplanar reformatting (MPR) technique on a workstation.

### Image analysis

Two radiologists (with 9 and 10 years of experience) reviewed the CT images and agreed by consensus. The thoracic CT scans of all patients were analyzed. In addition, 26 RT-PCR (+) and 13 RT-PCR (−) patients treated according to the same protocol (favipiravir and/or chloroquine and supplemental anticoagulant agent) based on the treatment guideline recommended by the Turkish Ministry of Health were examined regarding changes in their CT images after the treatment. Follow-up CT scans were obtained 1 month (17 patients) and 2 months (22 patients) after the first CT scan according to the clinical conditions of the patients.

The lesion patterns were classified as pure GGO, GGO, and consolidation combinations (GGO + nodular consolidation, GGO + patchy consolidation, GGO + segmental consolidation), consolidation, crazy-paving pattern, subpleural curvilinear line, air bubble sign, halo sign, reverse halo sign, air bronchogram, airway changes, or fibrous stripes. The distribution patterns of the lung tissue lesions were classified as peripheral (outer one-third of the lung), central (inner two-thirds of the lung), or peripheral + central localization. Images were also evaluated in terms of bilateral distribution and affected lung lobes. In patients who underwent follow-up CT imaging, changes in comparison to the first CT image were classified as increased, unchanged, regressed, or normal.

### Statistical analysis

SPSS 22.0 for Windows was used for statistical analysis. The mean ± standard deviation values are given for numerical variables. The frequency of radiological findings and the number of occurrences in each cluster are expressed as percentages. Lesion patterns, lesion distributions, and distribution patterns in the groups were compared using the chi-square test, and *p* < 0.05 was considered statistically significant.

## Results

### Clinical findings

Of the 130 patients included in the study, 48 (36.9%) were female, and 82 (63.1%) were male. The mean age of all patients was 49.8 ± 16.0 years (range, 18–92 years).

A total of 94 of the 130 enrolled patients had clinical symptoms. Fever and cough were present in 43 patients (50.6%) in the RT-PCR (+) group and 31 patients (68.9%) in the RT-PCR (−) group. There were no clinical symptoms in a total of 36 patients. Of those, 25 (29.4%) were in the RT-PCR (+) group and 11 (24.4%) in the RT-PCR (−) group.

### Imaging findings

**Fig. 2:**
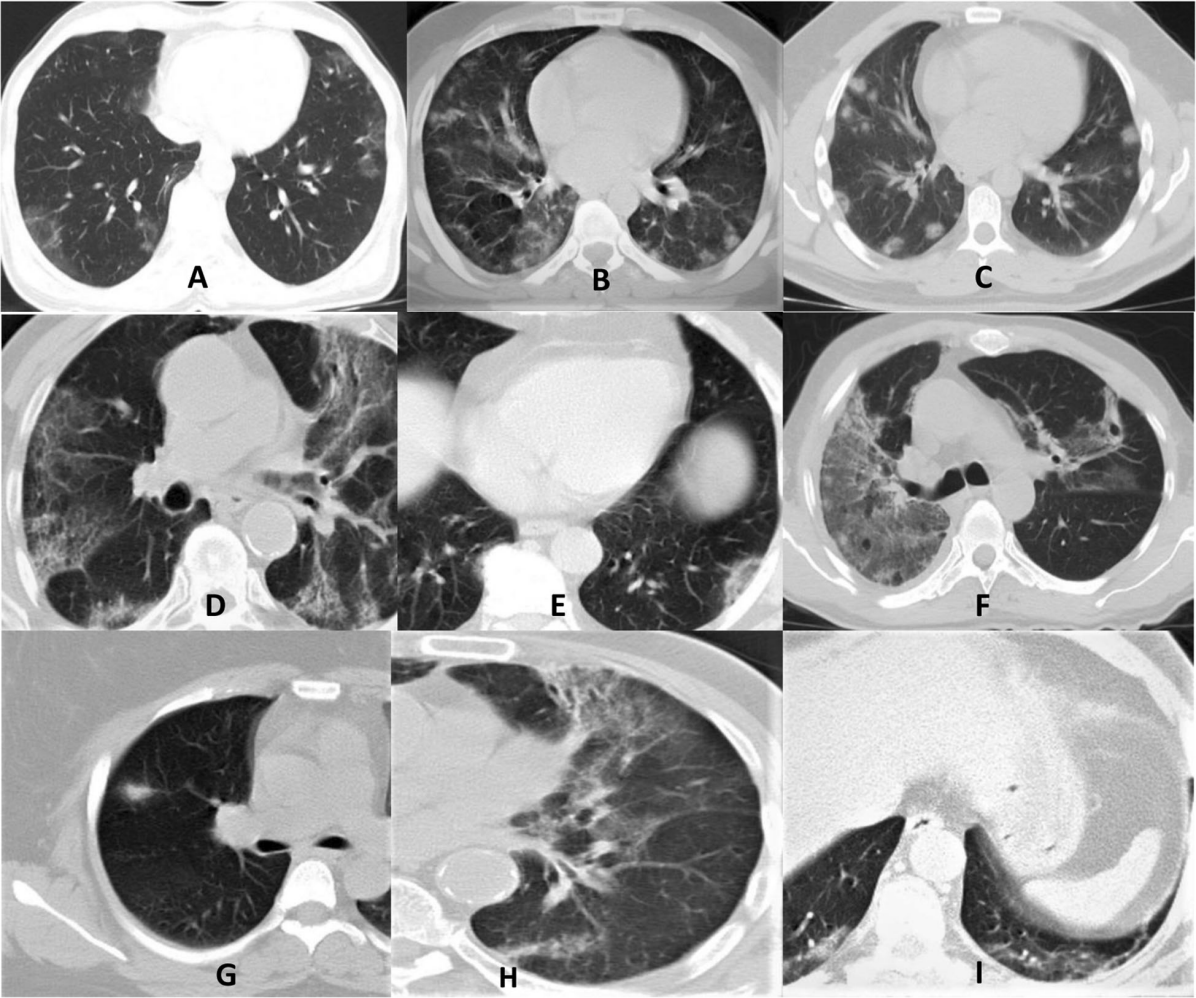
Lesion patterns on CT images of RT-PCR (+) patients. **a** GGO, **b** GGO + consolidation, **c** Nodular consolidation, **d** Crazy paving, **e** reversed halo sign, **e** air bubble sign, **g** halo sign, **h** airway changes, **i** curvilinear line

There was no statistically significant difference between the rates and types of lesion patterns observed in individuals with positive and negative test results (Fig. 2). GGO and GGO + consolidation (mixed) were the most common lesion patterns in both groups. In the RT-PCR (+) and RT-PCR (−) groups, bilateral (67.1% and 86.7%, respectively) and lower lobe (right 63.5% and 88.9%, left 74.1% and 88.9%, respectively) lesion distributions were most common. The most frequent distribution pattern was the peripheral distribution (61.2% and 55.6%, respectively). The lesion patterns, distributions of lesions, and distribution patterns seen in patients of both groups are shown in Table 1.

**Table 1:** CT imaging findings of patients (GGO; ground glass opacity, RT-PCR; reverse transcription polymerase chain reaction)

Group	RT-PCR (+) (*n* = 85)	RT-PCR (−) (*n* = 45)	*P*
Lesion patterns			
GGO	29 (34,1%)	22 (48.9%)	0.101
GGO + Consolidation			
Nodular	11 (12.9%)	8 (17.8%)	0.552
Patchy	26 (30.6%)	16 (35.6%)	0.565
Segmental	1 (1.2%)	3 (6.7%)	0.850
Consolidation	11 (12.9%)	10 (22.2%)	0.171
Crazy paving pattern	3 (3.5%)	1 (2.2%)	0.681
Curvilinear line	8 (9.4%)	11(24.4%)	0.021
Air bubble sign	2 (2.4%)	1 (2.2)	0.962
Halo sign	3 (3.5%)	0 (0.0%)	0.202
Reversed halo sign	3 (3.5%)	1 (2.2)	0.681
Air bronchogram	9 (10.6%)	6 (13.3%)	0.641
Airway changes	2 (2.4%)	2 (4.4%)	0.511
Fibrous stripes	21 (24.7%)	12 (26.7%)	0.807
Distribution pattern			
Peripheral	52 (61.2%)	25 (55.6%)	0.535
Central	1 (1.2%)	0 (0.0%)	0.465
Peripheral + Central	15 (17.6%)	20 (44.4%)	0.001
Distribution			
Bilateral	57 (67.1%)	39 (86.7%)	0.016
Right upper lobe	39 (45.9%)	34 (75.6%)	0.01
Right middle lobe	31 (36.5%)	27 (60.0%)	0.10
Right lower lobe	54 (63.5%)	40 (88.9%)	0.02
Left upper lobe	41 (48.2%)	34 (75.6%)	0.03
Left lower lobe	63 (74.1%)	40 (88.9%)	0.048

**Fig. 3:**
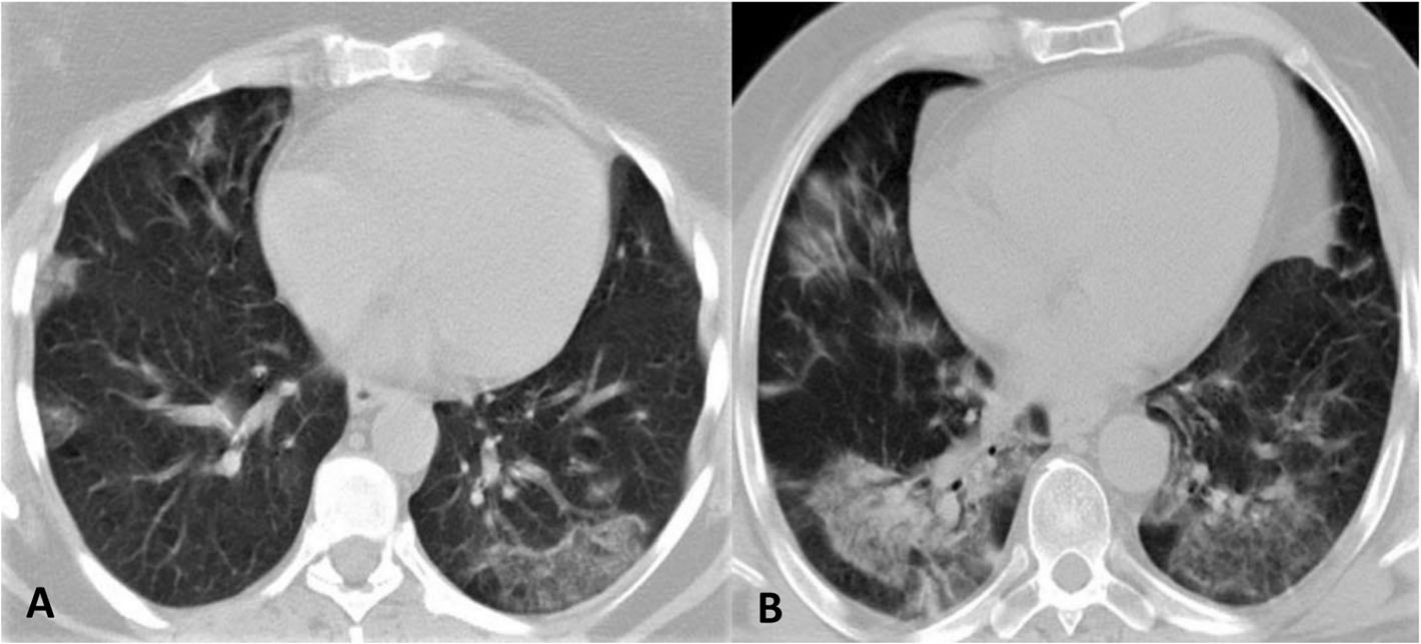
CT images of asymptomatic patients. **a** Axial CT image of a 50-year-old RT-PCR (+) asymptomatic female patient shows bilateral peripheral ground glass areas. **b** Axial CT image of a 52-year-old RT-PCR (−) asymptomatic male patient shows bilateral peripheral ground glass opacities, consolidation, and reticular opacities

Among asymptomatic patients, 15 RT-PCR (+) vs. 11 RT-PCR (−) patients had CT findings (Fig. 3). The most common lesion pattern in asymptomatic patients of both groups was GGO + patchy consolidation (46.7% and 63.6%, respectively). In these two groups, bilateral and lower lobe distributions were dominant (Table 2). All 26 asymptomatic patients from both groups had a peripheral (61.5%, 16 patients) or a peripheral + central (38.5% of 10 patients) distribution as the distribution pattern; thus, an exclusively central distribution was not observed.

**Table 2:** CT imaging findings of asymptomatic patients (GGO; ground glass opacity, RT-PCR; reverse transcription polymerase chain reaction)

Group	RT-PCR (+) (*n* = 15)	RT-PCR (−) (*n* = 11)
Lesion patterns		
GGO	4 (26.7%)	2 (18.2%)
GGO + Consolidation		
Nodular	3 (20.0%)	2 (18.2%)
Patchy	7 (46.7%)	7 (63.6%)
Segmental	0 (0.0%)	1 (9.1%)
Consolidation	2 (13.3%)	1 (9.1%)
Crazy paving pattern	1 (6.7%)	0 (0.0%)
Curvilinear line	1 (6.7%)	4 (36.4%)
Air bubble sign	0 (0.0%)	0 (0.0%)
Halo sign	1 (6.7%)	0 (0.0%)
Reversed halo sign	1 (6.7%)	1 (9.1%)
Air bronchogram	2 (13.3%)	3 (27.3%)
Airway changes	0 (0.0%)	2 (18.2%)
Fibrous stripes	1 (6.7%)	3 (27.3%)
Distribution		
Bilateral	11 (73.3%)	11 (100%)
Right upper lobe	5 (33.3%)	10 (90.9%)
Right middle lobe	4 (26.7%)	8 (72.7%)
Right lower lobe	10 (66.7%)	11 (100%)
Left upper lobe	7 (46.7%)	11 (100%)
Left lower lobe	14 (93.3%)	11 (100%)

Among patients of both groups who underwent a follow-up CT scan in the first month, 9 of 17 patients presented disease regression in their CT findings. In seven of these patients, the consolidations seen on the first CT were completely resorbed, and there was a decrease in the size and density of GGOs. Five of these patients had newly developed fibrous stripes. In the other two patients showing regression, there was a decrease in consolidations and GGOs.

The images of five patients with follow-up CT scan in the first month were normal, and there was no significant change in the images of two patients. One patient in the RT-PCR (−) group had an increase in initial CT findings (both GGOs and consolidations), and this patient had a history of chronic lymphocytic leukemia. One of the two patients in the RT-PCR (−) group without significant changes in the follow-up CT examination had a history of diabetes mellitus, chronic renal failure, and hypertension.

**Fig. 4:**
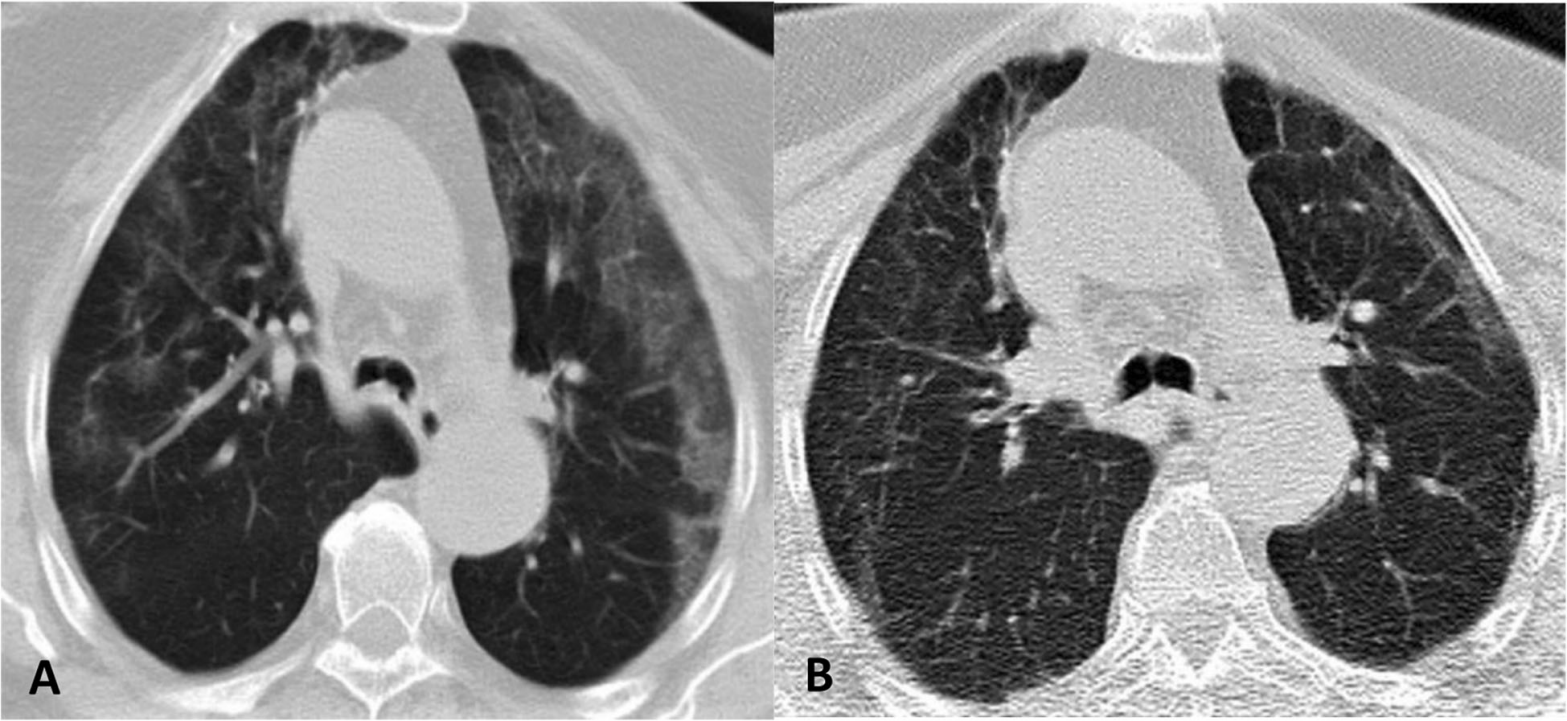
A 55-year-old RT-PCR (+) female patient with fever, cough, and abdominal pain. **a** Initial CT image obtained shows bilateral peripherally located ground glass areas. **b** 2nd month follow-up CT image shows almost complete regression of ground glass opacities and shows newly developed fibrous stripes

In 19 RT-PCR (+) patients who underwent a follow-up CT scan in the second month, one patient had an increase in CT findings, and eight patients showed signs of regression (Fig. 4). There was no change in one patient, and CT images were normal in nine patients. While one of the three RT-PCR (−) patients presented regression, two patients had normal CT findings

The consolidations were completely resorbed in nine patients from both groups who presented regression in the second-month follow-up CT images, and there was a significant reduction in the size and density of GGOs. One patient did not have the crazy-paving pattern observed in the first CT image. One RT-PCR (+) patient who showed an increase in number and severity of findings on the second-month follow-up CT scans had new consolidations. This patient had a history of Wegener granulomatosis. There was no known comorbidity in the one RT-PCR (+) patient with no significant CT changes over time.

## Discussion

Although RT-PCR test is considered the gold standard for definitive diagnosis, it has limitations such as detection sensitivity of COVID-19, long waiting time for results, staff experience, varying test protocols between countries, and quality differences of kits. Serial RT-PCR tests are performed to avoid these limitations, but this prolongs the diagnostic time. In addition, insufficient test kit resources can prevent retesting. Studies comparing the diagnostic accuracy of RT-PCR tests and CT findings in COVID-19 disease reported that the RT-PCR test may show false-negative results; the sensitivity of the test varies between 50 and 83%. Moreover, some studies suggest that the sensitivity of CT findings is higher than that of RT-PCRs [17–19]. In a study conducted by Xie et al. with 167 patients, it has been reported that all 5 patients with negative RT-PCR and positive CT at initial presentation were in direct contact with COVID-19 patients or that there were COVID-19 patients in their family. In the repeated RT-PCR tests, the positivity time varying between 2 and 8 days was detected in these patients [20].

In a study by Ai et al., 1014 patients were examined and CT findings were detected in 308 patients with negative RT-PCR results. Bilateral lung lesions consisting of ground glass opacities and consolidations were detected in lung CT of these 308 patients. For patients with a follow-up RT-PCR test, the mean interval between the initial negative to positive RT-PCR results is reported as 4–8 days. In this study, when RT-PCR results were taken as the reference standard, the sensitivity, specificity, and accuracy of chest CT in demonstrating COVID-19 infection were found to be 97%, 25%, and 68%, respectively [21].

In this retrospective study, we compared the clinical symptoms and CT findings of RT-PCR (+) and RT-PCR (N) patients. Both groups showed similar symptoms with fever and cough being the most frequent symptoms.

In our study, 80% of patients confirmed to have COVID-19 with RT-PCR assays showed positive findings at chest CT and typical CT findings were present in all patients with negative RT-PCR tests. In their review of 45 studies involving 4410 patients, Ojha et al. reported that the most common lesion patterns were GGO, consolidation, and GGO + consolidation (mixed) [22]. These findings were mostly observed in bilateral, peripheral, and lower lobe distributions. Similar results have been reported in different studies, and other CT findings such as crazy-paving pattern, halo and reversed halo sign, air bubble sign, subpleural curvilinear line, air bronchogram, airway changes, and fibrous stripe formations have been described [13, 14, 15]. Similar to these findings in the literature, GGO, GGO + consolidation, and consolidation were the most common lesion patterns in both groups examined in our study. Other findings identified were less frequent in both groups, and fibrous stripes were mostly seen on follow-up CTs at 1 and 2 months. Moreover, in accordance with the literature, bilateral, peripheral, and lower lobe lesion distributions were more frequent.

The COVID-19 literature reports that GGOs generally appear in follow-up CTs within the first 5 days after symptom onset, and the peak level is reached approximately 6–14 days after the onset of symptoms (GGO + consolidation). After 14 days, the consolidations regress more significantly, and resolution or fibrosis occurs toward the fourth week [22–24]. In our study of patients who underwent the same treatment protocol, resorption of the consolidations and a significant decrease in GGOs were detected in both groups in the first- and second-month follow-up CTs, and new fibrotic changes were observed in some patients. These findings were similar in both groups. Follow-up CT images were completely normal in 16 patients.

Our study identified 26 asymptomatic patients with CT findings. The frequency and distribution of lesion patterns in these patients of both groups were consistent with typical findings in the literature. Similar and lower viral loads have been reported in asymptomatic patients compared to symptomatic patients in previous studies [25, 26, 27]. There are insufficient data on the contagiousness of asymptomatic individuals and CT scanning may be helpful for the decision of isolation in clinically highly suspected asymptomatic RT-PCR (−) cases.

In studies examining the presence of ACE2 protein in samples taken from different tissues, high ACE2 protein expression in type 1 and type 2 alveolar epithelial cells but low expression in the cytoplasm of bronchial epithelial cells were observed. ACE2 protein was not detected in the nasal and oral mucosa or the surface epithelial cells of the nasopharynx. A study showed that BAL and tracheal aspirate samples had the highest viral load [28]. The upper respiratory tract’s positive RT-PCR test results may have been caused by the lower respiratory tract [29]. In individual patients, COVID-19 disease shows different viral load kinetics; thus, sampling timing, test quality, and disease progression have a significant impact on RT-PCR test results [30].

In this study, the laboratory findings, clinical symptoms, CT findings, and changes in CT findings after treatment were similar for COVID-19 patients and RT-PCR (−) patients with clinically suspected severe acute respiratory syndrome coronavirus 2 (SARS-CoV-2) infection. Specifically, the frequencies, types, distributions, and distribution patterns of lesions on CT scans were similar in both groups.

In our study, no positivity was observed in the repeat tests of patients in the RT-PCR (−) group, and therefore the diagnosis of COVID-19 could not be made definitively. However, all patients in this group had contact with the COVID-19 patient or a recent history of travel abroad, and all of these patients also had typical CT findings. For these reasons, these patients are highly suspicious of COVID-19 disease. RT-PCR test may not be positive in these patients due to reasons such as incomplete sampling techniques, variations in viral load, sampling time after contact, late transfer to the laboratory, and kit sensitivity.

This study has some limitations. First of all, the sensitivity of RT-PCR test kits may be low. Second, tracheal aspirate and BAL sampling could not be performed in RT-PCR (−) patients due to high transmission risk. According to various studies with larger study populations, BAL samples of patients with negative nasopharyngeal PCR results should be re-tested. Third, the number of patients with follow-up CT scans was small.

## Conclusion

In conclusion, RT-PCR test may show false negativity due to various reasons. Studies have shown that chest CT has a high sensitivity for the diagnosis of COVID-19. A patient with high clinical suspicion of COVID-19 infection with a history of contact should not be removed from isolation without a CT scan, even if RT-PCR tests are negative. If patients with negative RT-PCR tests but positive CT findings are discharged without isolation or other precautionary measures, the rates of human-to-human transmission may increase, and the patients may deteriorate. Chest CT can serve as a superior screening tool to RT-PCR in case of resource shortages in tests.

## Abbreviations

CT: Computed tomography
COVID-19: Coronavirus disease 2019
RT-PCR: Reverse transcription polymerase chain reaction
2019-nCoV: Novel coronavirus
SARS-CoV 2: Severe acute respiratory syndrome coronavirus 2
BAL: Bronchoalveolar lavage
ACE2: Angiotensin-converting enzyme 2
GGO: Ground-glass opacity
SARS-CoV: Severe acute respiratory syndrome coronavirus
MERS-CoV: Middle East respiratory syndrome coronavirus
